# Gate Tuning of Synaptic Functions Based on Oxygen Vacancy Distribution Control in Four-Terminal TiO_2−x_ Memristive Devices

**DOI:** 10.1038/s41598-019-46192-x

**Published:** 2019-07-10

**Authors:** Zenya Nagata, Takuma Shimizu, Tsuyoshi Isaka, Tetsuya Tohei, Nobuyuki Ikarashi, Akira Sakai

**Affiliations:** 1 Graduate School of Engineering Science, Osaka University, 1-3 Machikaneyama-cho, Toyonaka, Osaka 560-8531 Japan; 2 Institute of Materials and System for Sustainability, Nagoya University, Furo-cho, Chikusa-ku, Nagoya 464-8603 Japan

**Keywords:** Electronic devices, Information storage

## Abstract

Recent developments in artificial intelligence technology has facilitated advances in neuromorphic computing. Electrical elements mimicking the role of synapses are crucial building blocks for neuromorphic computers. Although various types of two-terminal memristive devices have emerged in the mainstream of synaptic devices, a hetero-synaptic artificial synapse, *i.e*., one with modulatable plasticity induced by multiple connections of synapses, is intriguing. Here, a synaptic device with tunable synapse plasticity is presented that is based on a simple four-terminal rutile TiO_2−x_ single-crystal memristor. In this device, the oxygen vacancy distribution in TiO_2−x_ and the associated bulk carrier conduction can be used to control the resistance of the device. There are two diagonally arranged pairs of electrodes with distinct functions: one for the read/write operation, the other for the gating operation. This arrangement enables precise control of the oxygen vacancy distribution. Microscopic analysis of the Ti valence states in the device reveals the origin of resistance switching phenomena to be an electrically driven redistribution of oxygen vacancies with no changes in crystal structure. Tuning protocols for the write and the gate voltage applications enable high precision control of resistance, or synaptic plasticity, paving the way for the manipulation of learning efficiency through neuromorphic devices.

## Introduction

Owing to significant developments in artificial intelligence technology, neuromorphic computing has attracted great attention in recent years. Electrical elements that mimic the role of synapses in the cerebral neural circuit are crucial building blocks for neuromorphic computers, and a memristive device is one of the promising candidates^1–10^. Much effort has been devoted to artificial synapse implementation using memristive devices, which typically have a metal-insulator-metal (MIM) structure with two electrode terminals, where the insulator is a metal oxide. Various kinds of mechanisms have been proposed to explain resistive switching (RS) phenomena in memristive devices. These vary depending on the MIM structure and operating conditions for the devices^11–18^. However, one commonly observed mechanism is based on dopant redistribution, which is often induced by the external voltage applied to the devices^19–24^. It is well known that oxygen vacancies act as donors in reduced TiO_2_ or nonstoichiometric TiO_2−x_. Thus, precise control of the oxygen vacancy distribution is essential for obtaining high-performance TiO_2−x_-based memristive devices.

Although the two-terminal structure is in the mainstream of synaptic devices, it is still challenging to develop an artificial synapse with a hetero-synaptic nature, *i.e*., modulatable plasticity induced by multiple connections of synapses^25–32^. Recently, we fabricated a novel type of memristive device on a rutile TiO_2−x_ single-crystal substrate^33^. This device had a simple four-terminal planar structure in which a pair of diagonally opposed electrodes was used to modify the distribution of oxygen vacancies in the electrically active zone of the device between another pair of diagonally opposed electrodes. A one-to-one correspondence was shown to exist between the morphologically controlled oxygen vacancy distribution in the device and the electrically controlled RS behavior showing a low resistance state (LRS) and a high resistance state (HRS). Furthermore, our previous study^33^ suggested the feasibility of a device with multilevel resistance on the basis of modification of the oxygen vacancy distribution, which would allow continuous weighting of synaptic devices for neuromorphic computing.

Here, we report RS results for four-terminal memristive devices under different applied voltage protocols, together with the microscopically analyzed causes for resistance changes in the devices. In addition, gate effects on both the RS properties and synaptic characteristics of the hetero-synaptic device are investigated thoroughly.

## Results and Discussion

**Figure 1:**
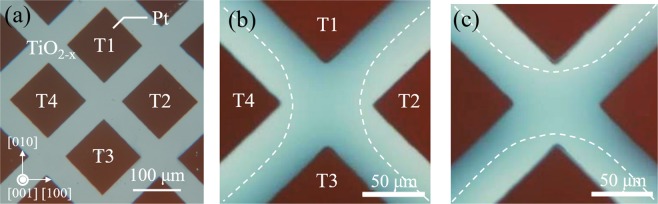
Optical micrograph of the device. (**a**) Optical micrograph of four-terminal device fabricated on reduced rutile TiO_2−x_ (001) surface. (**b**,**c**) Optical micrograph of TiO_2−x_ four-terminal device after positive voltage application (b) and negative voltage application (**c**). Colored region indicated by dotted lines where oxygen vacancies have accumulated can be controlled by positive and negative *V*_2,4_ application.

A four-terminal planar device with two pairs of diagonally opposed Pt electrodes formed on a TiO_2−x_ reduced crystal is shown in the transmission-mode optical micrograph in Fig. 1(a). The terminals are labeled T1 to T4, with T2 and T4 being used to control the oxygen vacancy distribution in the region between T1 and T3. By exploiting electrocoloring phenomena^34,35^, we can visualize the oxygen vacancy distribution in TiO_2−x_, which affects the electrical properties of the memristive device: regions with a higher (lower) concentration of oxygen vacancies are colored (colorless) and more (less) conductive. Figure 1b and c show, respectively, optical micrographs of the device taken after positive and negative voltages *V*_2,4_ were applied to both T2 and T4 simultaneously, while T1 and T3 were grounded. In the case of the positive (negative) applied voltage, a colored region bridging T1 and T3 (T2 and T4) was observed, while the resistance between T1 and T3 decreased (increased). This is likely because positively charged oxygen vacancies are repelled from T2 and T4 (T1 and T3), and then accumulate around T1 and T3 (T2 and T4) owing to drift motion induced by the electric field under the positive (negative) voltage *V*_2,4_. Thus, depending on the morphology of the colored region, the device can be configured to be in a LRS or HRS, which correspond to the colored region bridging or separating T1 and T3, respectively. Thus, morphological control of the oxygen vacancy distribution enables a resistance change in the device.

**Figure 2:**
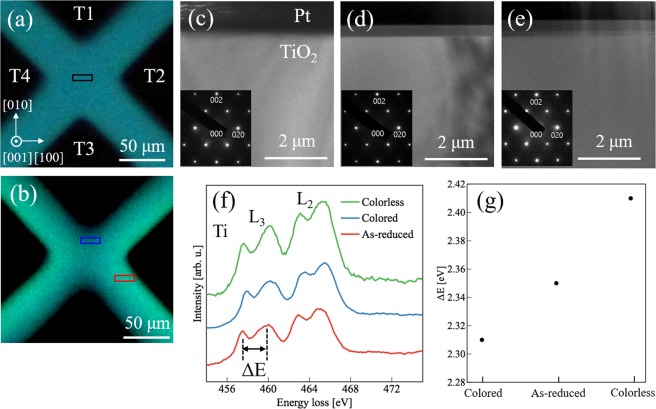
TEM and STEM-EELS analysis of the device. Optical micrograph of four-terminal device before (**a**) and after (**b**) voltage application. As-reduced, colored and colorless TEM and STEM-EELS samples were prepared from the regions indicated by black, blue, and red rectangles, respectively. (**c**–**e**) TEM images and corresponding ED patterns (inset) of as-reduced, colorless, and colored regions, respectively. No significant changes were observed between the TEM images and ED patterns, meaning that no crystal structural changes were involved in the device RS behavior. Note that observed Pt layers on TiO_2_ are protective layers for preparation of the cross-sectional TEM specimens and not the electrodes. (**f**) Ti L_2,3_ edge EELS spectra taken from several tens of nanometers from the surface of as-reduced (red curve), colored (blue curve) and colorless (green curve) regions in TiO_2−x_ four-terminal device. (**g**) e_g_-t_2g_ peak splitting Δ*E* of Ti L_3_ edge for three different regions. A decrease (increase) of Δ*E* in the colored (colorless) region indicates that the colored (colorless) region is in a reduced (oxidized) Ti valence state.

To elucidate the cause of the resistance change in the device, transmission electron microscopy (TEM) and scanning transmission electron microscopy-electron energy loss spectroscopy (STEM-EELS) analyses were performed. The cross-sectional TEM specimens for these analyses were prepared using a focused ion beam system from a portion of the device before and after applying *V*_2,4_ = 6 V for 50 s, as depicted in the optical micrographs of Fig. 2(a,b), respectively. Three different regions were selected for observation: an as-reduced region before voltage application (black rectangle in the figure), and a colorless (red) region and colored (blue) region after voltage application. Cross-sectional TEM images of all three regions are shown in Fig. 2(c–e) with the corresponding electron diffraction (ED) patterns (insets in the figures), respectively. No significant changes from the rutile crystal structure were observed in the TEM image or ED pattern for any region, which means that the reduction and applied voltage do not induce crystallographic modification in TiO_2−x_. This is in sharp contrast to the case of previously reported (100) TiO_2−x_ four-terminal memristive devices, where a magneli-like phase was formed in the colored region^36^.

We also analyzed the local electronic valence state of each RS region through STEM-EELS measurements conducted several tens of nanometers from the surface. Here, we mainly focus on EELS spectra with the Ti-L_2,3_ peak to analyze the Ti valence state in TiO_2−x_. The corresponding spectra are shown in Fig. 2f, and the energy splitting between the e_g_ and t_2g_ orbital peaks on the L_3_ edges (Δ*E*) for each region are shown in Fig. 2g. Note that Δ*E* is larger (2.41 eV) in the colorless region, and smaller (2.31 eV) in the colored region, as compared to that in the as-reduced region (2.35 eV). It is known that e_g_-t_2g_ orbital peak splitting is affected by the oxygen content in rutile TiO_2_^37–40^: when the octahedrally coordinated oxygen ions around Ti are deficient in reduced TiO_2_, the degree of splitting decreases. Thus, we infer that the colored (colorless) regions of the device are in a reduced (oxidized) Ti valence state. These Ti valence state changes, unaccompanied by any crystal structure changes, must be the origin of the durable RS behavior in the present device^33^.

**Figure 3:**
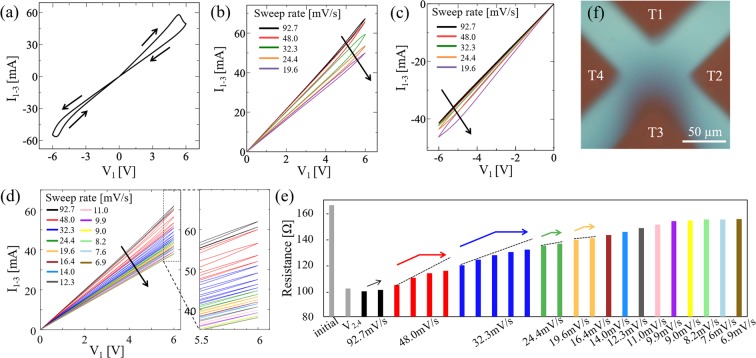
Electrical Characterization of the device. (**a**) *I-V* characteristics of device during voltage sweep with sweep rate 19.6 mV/s. (**b**,**c**) *I-V* characteristics of device during consecutive positive and negative voltage sweeps, respectively. Each line show hysteresis curves obtained by setting the voltage sweep rate *V*_1_ to 92.7, 48.0, 32.3, 24.4, and 19.6 mV/s. (**d**) *I-V* characteristics of device during consecutive positive voltage sweeps. Further multilevel resistance was achieved by repeating the sweep at a constant sweep rate between 92.7 and 6.9 mV/s. (**e**) Variation of resistance between T1 and T3 after each voltage application. Although the resistance was observed to saturate when a voltage sweep with the same sweep rate was applied (see dotted line as a guide to the eye), a further increase in resistance can be achieved by lowering the sweep rate. (**e**) Optical micrograph of device taken in the last stage of the measurement. Oxygen vacancies accumulated near T3 show as dark contrast.

RS characteristics were also measured by sweeping the voltage *V*_1_ applied to T1, with T3 grounded, and by applying *V*_2,4_ as a gate voltage. First a voltage of *V*_2,4_ = 6 V was applied for 50 s and typical *I-V* hysteresis curve is shown in Fig. 3(a), which was measured with a sweep rate of 19.6 mV/s. To demonstrate the multilevel resistance modulation, five consecutive positive voltage sweeps of *V*_1_ were conducted with stepwise decreased sweep rates (92.7, 48.0, 32.3, 24.4, and 19.6 mV/s). Then, five consecutive negative voltage sweeps of *V*_1_ were conducted with the same stepwise decreased sweep rates. The *I-V* curves of each positive and negative sweep are shown in Fig. 3(b,c), respectively. Each *I*-*V* curve clearly exhibits hysteresis, indicating RS from LRS to HRS for positive sweeps and HRS to LRS for negative sweeps. The current value gradually decreased through repeated *V*_1_ sweeping in which the sweep rate was decreased step by step. The resistance gradually increased (decreased), showing multilevel values as the *V*_1_ sweeping process was repeated. These RS properties may be attributable to the drift of oxygen vacancies, and the presumed scenario is that oxygen vacancies are repelled from T1 when a positive voltage is applied and drawn back to T1 when a negative voltage is applied.

Next, the dependence of the RS properties on the voltage sweep count was also examined. In this operation, starting with a high sweep rate, *V*_1_ voltage sweeping was repeated several times at a constant sweep rate. *I-V* curves obtained by extensive positive voltage sweeping and the corresponding resistance change are shown in Fig. 3(d,e), respectively. By combining repetitive sweeps and a gradual reduction in the sweep rate, we obtained very fine multilevel resistance states. An optical microscope image taken after the final sweep is shown in Fig. 3(f). The dark region around T3 and the colorless region around T1 indicate that the oxygen vacancies were repelled from T1 and accumulated around T3 as a result of the consecutive voltage sweeps. This oxygen vacancy distribution accounts for the observed resistance increase.

It is also observed in Fig. 3(e) that the resistance increase tends to saturate after several sweeps for all sweep rate but lowering the sweep rate brings further resistance increase. This can be explained by the drift observed in oxygen vacancies when consecutive voltage sweeps were conducted at a constant sweep rate. In the present case, the state variable *w*, which determines the resistance of the device, represents the effective length of the region with a low concentration of oxygen vacancies having high resistance in the electrically active region between T1 and T3. Since the time derivative of *w* is considered to be proportional to the current flow in the memristor^2,24^, the reduction in current due to the resistance increase restrains the elongation of *w*, saturating the effects of heightening the resistance. On the other hand, slowing the sweep rate substantially increases the time integral of the current flow, *i.e*., the total charge passing through the device per sweep cycle. This mechanism recovers the elongation of *w*, allowing the resistance to increase further.

Next, we explore the possibility of modulating the RS characteristics of our device for potential use as an artificial synapse with tunable plasticity. The potentiation and depression characteristics of a modulatable synaptic device are determined using applied voltage protocols consisting of write pulse, gating pulse, and non-disturbing read pulse steps. More specifically, *V*_2,4_ is applied to accumulate oxygen vacancies between T1 and T3. Then, consecutive positive write pulses *V*_write_ are applied to T1 until the device resistance reaches a target value in HRS for the depression process. Subsequently, consecutive negative write pulses are applied to T1 until the resistance reaches a target resistance value in LRS for the potentiation process. In addition, a gating pulse of amplitude *V*_gate_ is applied to both T2 and T4 simultaneously with or prior to each write pulse during depression or potentiation, respectively. This sequence is repeated until the device function fails, where failure is defined as the point at which the number of pulses required to reach the HRS (LRS) deviates by 1.5 times the interquartile range from the upper (lower) limit of the interquartile range.

In the present experiments, failure usually occurred when the resistance could no longer be reduced by application of a negative *V*_write_ (see Supplementary Fig. S1). To elucidate the possible cause of this phenomenon, the effects of consecutive negative voltage sweeping on the device resistance were examined. In the initial stage of RS from HRS to LRS, each voltage sweep caused a further decrease in resistance. However, in the later stage, we tended to encounter an unexpected resistance increase despite the negative voltage application (see Supplementary Fig. S2(a,b)). Inspection of the device by optical microscopy revealed that the dark region between T1 and T3 became blurred compared with that for the device in the lower resistance state (see Supplementary Fig. S2(c,d)). In the case of a negative applied voltage, the electric field is directed from T3 to T1, making oxygen vacancies around T3 drift towards T1. This is expected to lower the resistance owing to the decrease of *w*; however, in the later stage, the oxygen vacancies rather tend to spread out, which dilutes the concentration in the device’s active region. We conclude that this distribution of oxygen vacancies caused the observed resistance increase and likely triggered the failure of the device. Given this mechanism, the application of gate voltage *V*_2,4_ is effective at confining oxygen vacancies to the region between T1 and T3. In fact, we confirmed that gating during potentiation can help to increase the endurance of depression/potentiation cycles markedly as compared to the case of no applied voltage (see Supplementary Fig. S3(a,b)).

**Figure 4:**
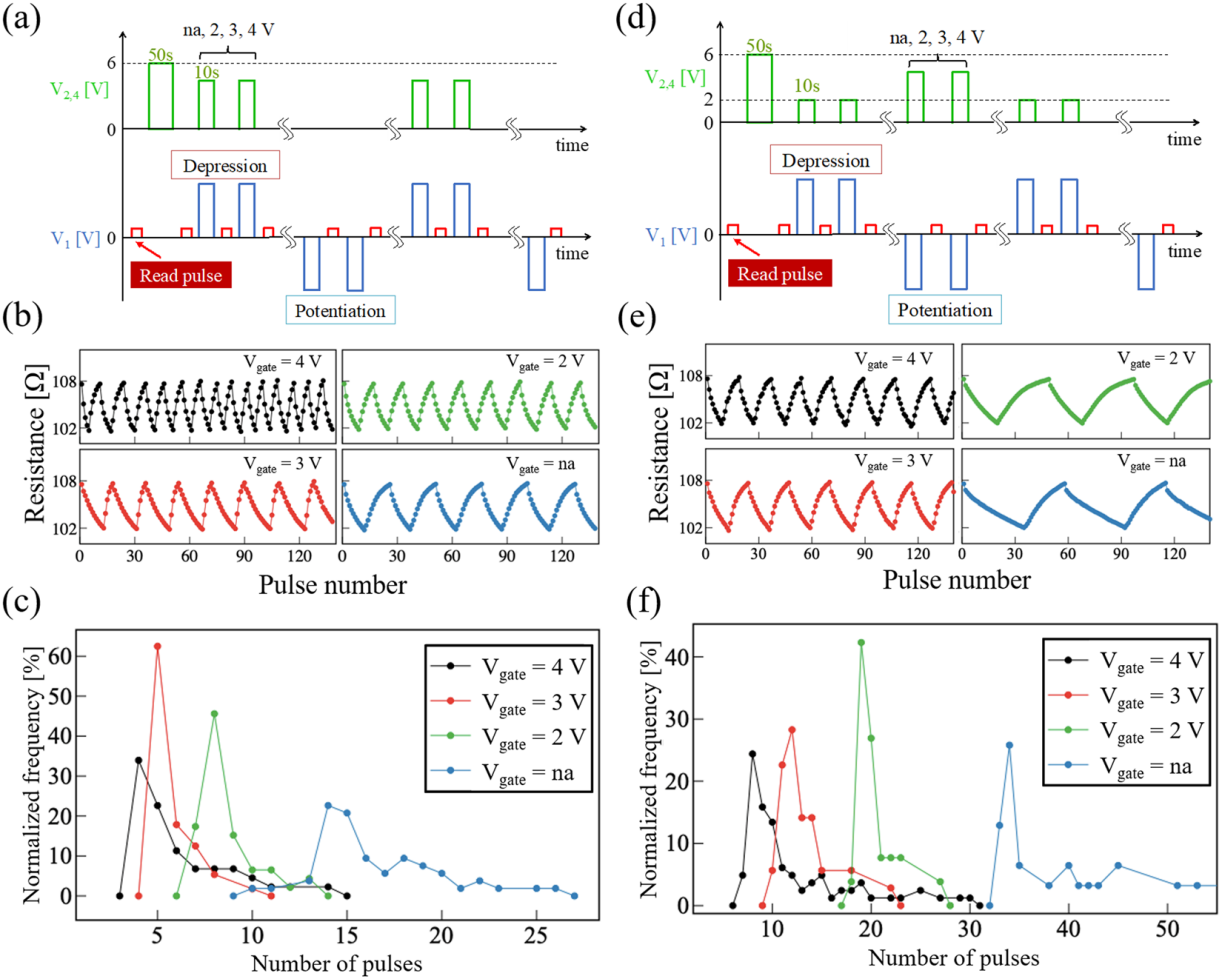
Modulation of the RS characteristics using gate voltage. (**a**,**d**) Voltage application protocol used in the measurement. Gate, write and read pulse are represented by green, blue, and red lines, respectively. (**b**,**e**) Potentiation and depression of the device with gate voltages of 4, 3, 2 V and na, indicated by black, red, green, and blue lines and dots, respectively. (**c**,**f**) Normalized frequency of the number of pulses required to reach the target HRS (107.5 Ω) or LRS (102 Ω) with gate voltages of 4, 3, 2 V and na, indicated by black, red, green and blue lines and dots, respectively.

One of the detailed protocols of the voltage application for gating during depression is shown in Fig. 4(a). First, a *V*_2,4_ = 6 V was applied for 50 s, and then a series of positive write pulses *V*_write_ = 4 V was applied to T1 until the device resistance reached the target value of 107.5 Ω for the depression process. Subsequently, a series of negative write pulses *V*_write_ = −4 V was applied to T1 until the resistance reached the target resistance value of 102 Ω for the potentiation process. Here, we set the target resistance ratio to be low in order to meet a sufficient endurance of potentiation/depression cycles. A gating pulse of amplitude *V*_gate_ was simultaneously applied when a positive *V*_write_ was applied during depression. Figure 4(b) shows the resistance modulation of the device with gate voltages, *V*_gate_, of 4, 3, 2 V and non-applied (na) in the protocol shown in Fig. 4(a). The cycle of resistance change depending on the pulse number is observed to vary systematically with the gate voltage. The gate voltage dependence of the number of pulses required for reaching the target HRS during depression is shown in Fig. 4(c). Note that the required number of pulses can be modulated by varying the applied gate voltage: the higher the gate voltage, the smaller the number. Thus, the depression rate as a synaptic device is tunable by the synchronized application of the gate voltage.

We also investigated the gate effect during potentiation by applying various gate voltages right before the application of each negative write voltage pulse *V*_write_. Figure 4(d) shows one example of an applied voltage protocol; the *V*_write_ during depression (potentiation) was set to +4.2 V (−4.2 V) and the gate pulse during depression was fixed at 2 V while that during potentiation was systematically varied. Figure 4(e,f) show, respectively, the resistance modulation and the number of pulses required for reaching the target LRS during potentiation with various gate voltages, *V*_gate_, of 4, 3, 2 V and na. It is found that gate pulse application during potentiation has the same effect on synaptic plasticity as that during depression: the higher the gate voltage, the smaller the number of required pulses. These results demonstrate that gate tuning of both the depression and potentiation properties is feasible in our four-terminal memristive device.

The above results for the gate effect can be understood by focusing on the oxygen vacancy distribution in the active region of the device. The application of a positive gate voltage induces a distribution of oxygen vacancies more concentrated in the region between T1 and T3 as compared with the case where no voltage is applied. Such an oxygen vacancy distribution gives rise to a larger resistance change per write pulse since the rate of resistance change increases with dopant concentration. Thus, at higher gate voltages, a larger modulation of the oxygen vacancy distribution is achieved, and fewer write pulses are required to reach the target HRS or LRS. This demonstrates that a pair of electrode terminals for gating can mimic the function of a neuromodulator and regulate the plasticity of hetero-synaptic synapses.

Although we have focused on rather ‘static’ RS behavior in the present paper, several dynamic functions such as spike-timing-dependent potentiation (STDP) or paired pulse facilitation (PPF) should also be important features for synaptic devices. We consider that implementation of such synaptic functions is possible by tuning voltage application protocols, i.e., switching polarities and (de)synchronization of input voltage pulses, for the electrodes in the four-terminal device, or by employing materials showing time-dependent relaxation behavior (or limited time retention) of resistive change state. Investigation of such dynamic synaptic behaviors should be an important subject for our future works. Concerning the power consumption, the present device shows measured current of several tens of milliamperes which is not a trivial value. To this point, preparing higher resistance TiO_2−x_ crystals and/or down-scaling the device size can help reduce the power consumption. Recently we have been preparing epitaxially-grown thin-film based devices where electronic active regions are confined to several tens of nanometers thickness, and observed that the device shows more than an order of magnitude higher resistance and lower current. Lateral length scale of the device can also be reduced down to a sub-μm scale by employing fabrication processes such as electron beam lithography. Following the rule of constant electric filed scaling, we expect that such down-sizing of devices should lead to significant reduction of power consumption. Also in the preliminary results of thin-film based devices, we observed that resistance ratio between HRS and LRS can be increased to more than 10 (>1000%), which provides enough weight modulation range in neural network applications. Finally, our concept of four-terminal memristive device can be extended into three dimensional (3D) integration for higher density implementation. Prototypic design of 3D four-terminal devices based on cross-bar type architecture is currently under investigation.

## Summary and Conclusions

In this work, four-terminal TiO_2−x_ single-crystal memristive devices were fabricated, and their RS characteristics and gating effects were determined. Through precise control of the oxygen vacancy distribution, stable RS operation and fine multilevel resistance modulation can be achieved by tuning the voltage application protocol, even with devices having a simple structure. An electronic change in the Ti valence state, rather than any change in crystallographic structure, is the crucial mechanism behind the RS in the present devices. The application of a gate voltage to the device, in conjunction with the write operation, effectively tunes the potentiation/depression behavior, *i.e*., the synapse plasticity of the device. The present approach opens the way to developing artificial synapses that exhibit hetero-synaptic properties with multiple connections of synapses and achieving very complex functions similar to those of a neuromodulator in a biological synapse.

## Methods

A non-doped rutile TiO_2_ (001) single crystal (10 × 10 × 0.5 mm) was reduced by thermal annealing at 700 °C for 6 h under a vacuum of 1.5 × 10^−6^ Pa to generate oxygen vacancies in the crystal. The resistivity of the thermally annealed samples was measured by the van der Pauw method and estimated to be about 6 Ω·cm. The Pt electrode deposition was carried out using magnetron sputtering through metal masks. Each electrode was confirmed in advance to have an ohmic contact with the substrate, which typically occurs for highly doped semiconductors.

All electrical measurements were performed using a tungsten needle prober station inside a vacuum chamber at a base pressure below 10^−3^ Pa and a Keysight B1500A semiconductor device analyzer. The resistance of the device was determined from the current flowing between T1 and T3 under an applied voltage of 1 V to measure the resistance change due to consecutive voltage application and under 100 mV to investigate the gate effects on synaptic plasticity.

In order to analyze the electronic and crystal structures in the electrically active region of the device, TEM and STEM-EELS analyses were carried out using a JEOL ARM-200F system at an accelerating voltage of 200 kV. EELS measurements were performed by using a Gatan Quantum spectrometer, and the spectra were recorded with a dispersion of 0.05 eV/channel. The energy resolution of EELS, *i.e*., the full width at half maximum of the zero-loss peak, was approximately 0.5 eV. The internal structure of the devices was inspected using an optical microscope (Olympus BH2-UMA) for visualizing the oxygen vacancy distribution in the device on the basis of the electrocoloring effect in TiO_2−x_.

## Supplementary information

Supplementary Information
