# Author Correction: Gate Tuning of Synaptic Functions Based on Oxygen Vacancy Distribution Control in Four-Terminal TiO_2−x_ Memristive Devices

**DOI:** 10.1038/s41598-019-51829-y

**Published:** 2019-10-17

**Authors:** Zenya Nagata, Takuma Shimizu, Tsuyoshi Isaka, Tetsuya Tohei, Nobuyuki Ikarashi, Akira Sakai

**Affiliations:** 1 Graduate School of Engineering Science, Osaka University, 1-3 Machikaneyama-cho, Toyonaka, Osaka, 560-8531 Japan; 2 Institute of Materials and System for Sustainability, Nagoya University, Furo-cho, Chikusa-ku, Nagoya, 464-8603 Japan

Correction to: *Scientific Reports* 10.1038/s41598-019-46192-x, published online 10 July 2019

This Article contains errors in the Results section.

“Each *I*-*V* curve clearly exhibits hysteresis, indicating RS from LRS to HRS for positive sweeps and HRS to LRS for negative sweeps. The current value gradually decreased through repeated *V*_1_ sweeping in which the sweep rate was decreased step by step. The resistance gradually increased (decreased), showing multilevel values as the *V*_1_ sweeping process was repeated.”

should read:

“Each *I*-*V* curve clearly exhibits hysteresis, indicating RS from LRS to HRS for positive sweeps and HRS to LRS for negative sweeps. The current value gradually decreased (increased) through repeated *V*_1_ sweeping in which the sweep rate was decreased step by step. The resistance gradually increased (decreased), showing multilevel values as the *V*_1_ sweeping process was repeated.”

