# Supplementary material for: Gate Tuning of Synaptic Functions Based on Oxygen Vacancy Distribution Control in Four-Terminal TiO2−x Memristive Devices
Source: Sci Rep. 2019 Jul 10;9:10013. doi: 10.1038/s41598-019-46192-x (PMC6620322; doi:10.1038/s41598-019-46192-x)
Supplement: Supplementary file 1 — Supplementary Information [file 41598_2019_46192_MOESM1_ESM.pdf]

Supplementary Information

Gate Tuning of Synaptic Functions Based on Oxygen Vacancy Distribution Control in Four-Terminal TiO_{2-x} Memristive Devices

Zenya Nagata,[1] Takuma Shimizu,[1] Tsuyoshi Isaka,[1] Tetsuya Tohei,[1,*] Nobuyuki Ikarashi,[2] and Akira Sakai[1,*]

Figure S1 shows representative results for the resistance transient during potentiation and depression of a device with gate voltages of 4, 3, 2 V and n/a, indicated by black, red, green and blue lines, respectively (partly shown in Fig. 4(b)). Note that, in all four cases, the device fails when the resistance could no longer reach the LRS through application of negative V_{write}.

To further investigate the cause of this phenomenon, consecutive negative voltage sweeping was conducted. The measured current $I_{1\text{-}3}$ between T1 and T3 as a function of the applied voltage V_1 is shown in Fig. S2(a), and the variation in resistance between T1 and T3 after each voltage application is shown in Fig. S2(b). Starting with a high sweep rate, negative voltage sweeps were repeated with a gradually decreasing sweep rate. The results clearly show a decrease in resistance owing to the negative voltage sweeps. However, after a negative voltage sweep with a rate of 14.0 mV/s, an increase is observed in resistance. We may understand the cause of this at the microscopic scale. Figure S2(c,d) show optical micrographs of the device after negative voltage sweeps of 16.4 and 14.0 mV/s, respectively. A comparison of these images shows that extensive negative voltage sweeping led to blurring of the dark contrast between T1 and T3, indicating an increase in resistance.

To examine the effect of gate voltage as a means of confining oxygen vacancies in the region between T1 and T3, the endurance of depression/potentiation cycles was investigated. Figure S3(a,b) show the number of depression/potentiation cycles to failure as a function of gate voltage during potentiation and depression, respectively. The write and gate voltage conditions for the three sets of gating during potentiation (GDP) and gating during depression (GDD) measurements are shown in Table S1 and S2, respectively. An increase in the number of cycles is clearly observed for the GDP case, while no remarkable increase is observed for the GDD case. From these results, we conclude that gate voltage application during potentiation is an effective way to confine oxygen vacancies between T1 and T3, which improves the endurance of the device.

Figures and figure captions

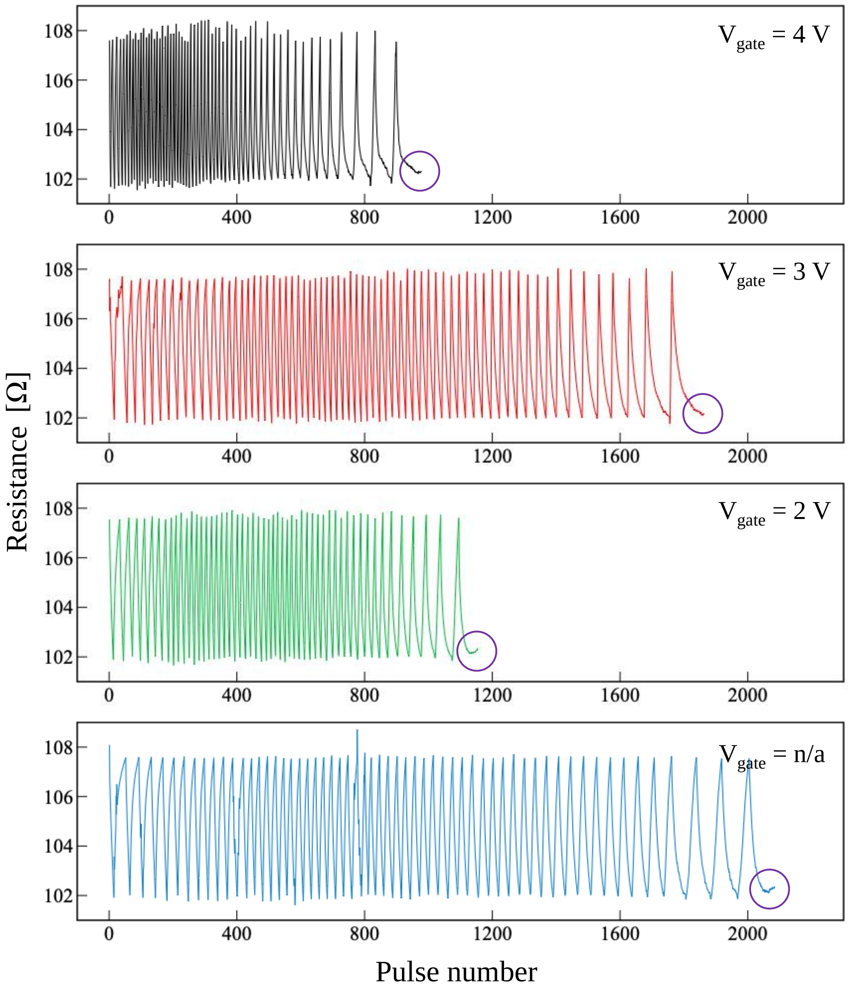

Figure S1. Typical data showing resistance transient of device during potentiation and depression processes, with gate voltages of 4, 3, 2 V and na indicated by black, red, green, and blue lines, respectively. In all cases, the device fails when the resistance can no longer be reduced, but starts to increase upon further negative voltage application (indicated by purple circles).

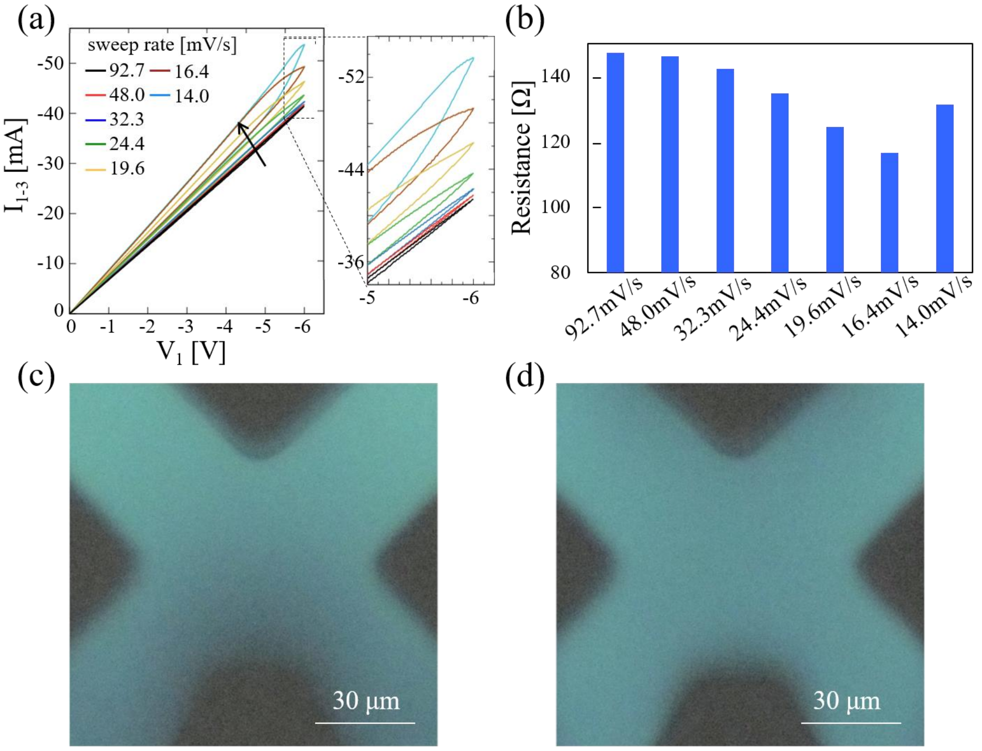

Figure S2. (a) *I-V* characteristics of device in HRS when consecutive negative voltage sweeps are applied. (b) Variation of resistance between T1 and T3 after each negative voltage application. (c)(d) Optical micrographs of devices after negative voltage sweeps of 16.4 and 14.0 mV/s, respectively.

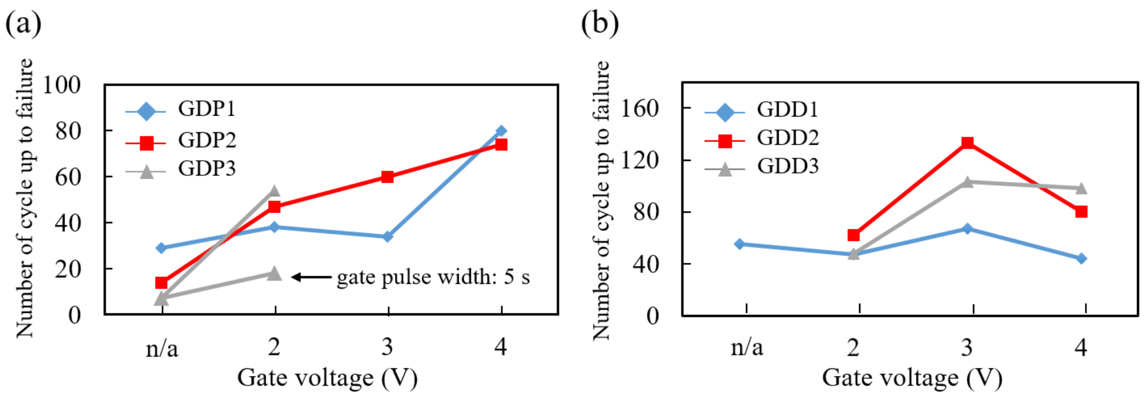

Figure S3. (a)(b) Number of depression/potentiation cycles to failure as function of gate voltage during potentiation and depression, respectively. The number of cycles tends to increase with gate voltage in the GDP case, while no such trend is observed in the GDD case. This shows that gate voltage application during potentiation is effective in confining the oxygen vacancies between T1 and T3, improving device endurance.

Table S1. Write and gate voltage conditions for GDP measurements shown in Figure S3a.

	Write voltage (V)	Gate voltage (V)	Gate pulse width (s)
GDP1	4.2	4.0	10
	4.2	3.0	10
	4.2	2.0	10
	4.2	n/a	0
GDP2	4.5	4.0	10
	4.5	3.0	10
	4.5	2.0	10
	4.5	n/a	0
GDP3	5.0	2.0	10
	5.0	2.0	5.0
	5.0	n/a	0

Table S2. Write and gate voltage conditions for GDD measurements shown in Figure S3b.

	Write voltage (V)	Gate voltage (V)	Gate pulse width (s)
GDD1	4.0	4	10
	4.0	3	10
	4.0	2	10
	4.0	n/a	0
GDD2	5.0	4.0	5.0
	5.0	3.0	5.0
	5.0	2.0	5.0
GDD3	5.0	4.0	5.0
	5.0	3.0	5.0
	5.0	2.0	5.0
